# Serum Relmβ combined with abdominal signs may predict surgical timing in neonates with NEC: A cohort study

**DOI:** 10.3389/fped.2022.943320

**Published:** 2022-09-06

**Authors:** Xiao-Chen Liu, Lu Guo, Ke-Ran Ling, Xiao-Yu Hu, Yu-Jie Shen, Lu-Quan Li

**Affiliations:** Neonatal Diagnosis and Treatment Centre of Children's Hospital of Chongqing Medical University, National Clinical Research Center for Child Health and Disorders, Ministry of Education Key Laboratory of Child Development and Disorders, China International Science and Technology Cooperation Base of Child Development and Critical Disorders, Chongqing Key Laboratory of Pediatrics, Chongqing, China

**Keywords:** abdominal signs, necrotizing enterocolitis, Relmβ, serum biomarkers, surgery

## Abstract

**Aims:**

To examine the predictive value of serum biomarkers combined with other indicators for necrotizing enterocolitis (NEC) surgery decision-making.

**Methods:**

Clinical data, including baseline information, clinical features, imaging presentation and serum assessment, of the infants enrolled were collected, and the serum concentrations of HBD2, HMGB-1, Claudin-3 and Relmβ were determined. *Student's t test*, the *Mann–Whitney U test*, the *chi-square test* and logistic regression analysis were used. Receiver operating characteristic (*ROC*) curves were also generated.

**Results:**

Forty-nine infants were enrolled, with 23 in the surgical NEC group and 26 in the medical NEC group. There were no differences in the baseline clinical information, including birth weight, gestational age, admission age and risk factors, during pregnancy and before enrollment (*P* > 0.05). Peritonitis, intestinal adhesion and sepsis were more common in the surgical group (*P* < 0.05). The incidences of abdominal distention, abdominal wall tenseness, abdominal tenderness and absent bowel sounds in the surgical group were significantly higher when NEC occurred (*P* < 0.05). There were no differences between the two groups in the imaging presentation (*P* > 0.05). The concentration of Relmβ {[8.66 (4.29, 19.28) *vs*. 20.65 (9.51, 44.65)]} in the surgical group was significantly higher (*P* < 0.05). Abdominal wall tenseness, abdominal tenderness and a Relmβ concentration > 19.7 μmol/L were included in the predictive model, and the AUC of the predictive score was 0.943 (95% CI: 0.891–1.000) (*P* < 0.05).

**Conclusion:**

Serum Relmβ concentration combined with abdominal wall tenseness and abdominal tenderness may be useful in determining surgical timing in neonates with NEC.

## Introduction

Necrotizing enterocolitis (NEC) is the most common and serious gastrointestinal inflammatory disease during the neonatal period with high morbidity and mortality. The incidence is 5–12% in preterm neonates and up to 13% in very low birth weight (VLBW) infants (1). Despite years of hard work, the fatality rate is still as high as 20–30% and can be even higher than 50% in those who have undergone surgeries due to inaccurate surgical indications (2, 3). Severe neurological retardation, enterostenosis, short bowel syndrome, cholestasis and other complications after unnecessary surgeries can affect the life quality of those infants subsequently (4, 5). Pneumoperitoneum on X-ray is an absolute surgical indication. However, by the time it is identified, the inflammation caused by intestinal perforation as well as septic shock or other fatal complications is life-threatening, and a surgical intervention can still not avoid death (6–8). Therefore, it is important to perform the surgery at the most suitable time. The ideal surgical indication is full-thickness intestinal necrosis but no perforation (5). It has been proposed that peritonitis signs, including abdominal tenderness, abdominal wall erythema, the absence of bowel sounds (9) and metabolic derangements, can predict intestinal inflammation before perforation (10). However, in regards to surgical decision-making, this indirect evidence has low sensitivity and does not reflect intestinal necrosis (5, 11). Thus, there seems to be a need for identifying indications related to intestinal necrosis.

Studies have shown that human β-defensin 2 (HBD2), high mobility group box-1 protein (HMGB-1), Claudin-3 and Resistin-like molecule β (Relmβ) are associated with intestinal inflammation (12–15). HBD2 is an antibacterial protein expressed during intestinal inflammation but not in normal epithelial tissues (14) and increases significantly in the feces of severe NEC patients (16). HMGB-1 is actively secreted by intestinal epithelial cells and can be released into the intestinal lumen by necrotic cells (15). It significantly increases in colitis and shows a constant upward-regulation trend with colitis aggravation (17). Claudin-3 is one of the most important tight junction proteins in the intestinal tract (13) and is increased in urine and intestinal tissue during intestinal inflammatory diseases (18, 19). Relmβ is a bactericidal protein produced by colonic epithelial goblet cells participating in the regulation of intestinal inflammatory diseases (20), and colitis mice with silenced Relmβ genes exhibit less mucosal injury (21). Luo found that the levels of serum Relmβ are significantly higher in neonates with NEC (22).

Based on previous studies, these biomarkers can reflect the severity of NEC disease to some extent (16–19, 22). However, few clinical studies have evaluated the predictive value of these biomarkers in surgical decision-making. We determined the serum concentrations of HBD2, HMGB-1, Claudin-3 and Relmβ by enzyme-linked immunosorbent assay (ELISA) and combined them with other clinical indicators to explore their predictive value in NEC surgery decision-making.

## Patients and methods

Neonates diagnosed with NEC at the Children's Hospital of Chongqing Medical University from March 2019 to October 2020 were included. Our research was approved by the Ethics Committee of the Children's Hospital Affiliated to Chongqing Medical University (No. 2019-284). All neonates enrolled in this trial had informed consent forms signed by their parents or guardians.

### Inclusion and exclusion

Neonates diagnosed with NEC [Bell stage II (23) or above according to laboratory results, ultrasound or radiography and based on clinical manifestations, such as abdominal distension, bloody stools, vomiting, fixed abdominal tenderness, absence of bowel sounds or abdominal wall tenseness] were enrolled in our study. Those who met the following criteria were excluded: (1) infants who needed surgery for pneumoperitoneum based on abdominal X-rays; (2) infants who were found to have less than full thickness necrosis in the surgery; (3) patients with other gastrointestinal diseases (such as congenital intestinal atresia, Hirschsprung disease, or intestinal malrotation), metabolic diseases, or other serious infections; (4) patients in whom a complete assessment of serum biomarkers was not obtained; and (5) patients for whom consent to participate in this study was not granted. There were no interventions to the infants enrolled.

### Grouping criteria

Neonates with fixed intestinal loops on imaging or significant clinical deterioration after pure medical treatment underwent surgery. The final surgical decisions were made by experienced senior surgeons. Those diagnosed with full-thickness intestinal wall necrosis but no perforation by pathological biopsy after surgery were included in the surgical group. Those who recovered after medical treatment were included in the medical group.

### Data collection

Relevant information, including baseline information, diseases and medication during pregnancy, delivery room handling, and hospital outcomes, was collected. Other clinical data, including abdominal signs and abdominal imaging data (both on X-ray and ultrasound) after the patients were diagnosed with NEC, were also presented.

### Serum sampling

The serum samples were collected in the two groups when the patients were diagnosed with NEC. The samples were immediately sent to the laboratory and stored in a freezer at −20°C for the detection of serum biomarkers. The concentrations of HBD2, HMGB-1, Claudin-3 and Relmβ were detected with ELISA kits.

### Biomarker detection

The serum samples were thawed and centrifuged at 3,000 rpm for 20 mins. Then, 50 μl supernatant samples were taken and mixed with 450 μl diluent in a 1.5 ml EP tube by shaking for 2 mins. Fifty microliters of the mixed sample was added to a 96-well microtiter plate in turn, and two duplicate wells were prepared for each sample. Then, the plate was sealed with the film and incubated at 37°C for 30 mins. After removing the sealing film, the wells were washed 5 times, 50 μl of enzyme-labeled reagent was added, and the plate was warmed in a bath and washed again. Fifty microliters of developer A and 50 μl of color developer B were added to each well in the dark. The washing solution was shaken and mixed, and then the samples were developed at 37°C for 15 min in the dark. Finally, 50 μl of stop solution was added to each well, and the absorbance was detected with a BioTeck Gen5 plate reader (Agilent, Santa Clara, CA, USA). A wavelength of 450 nm was set, and a blank well was used for zero adjustment. The standard concentration and the absorbance value were entered into Reader Fit software for analysis. The actual concentration was ten times the analyzed concentration, and the average value of two duplicate wells was taken for the final concentration.

### Data analysis

All statistics were analyzed with *SPSS* statistical software (version 24; SPSS, Chicago, IL, USA). Normally distributed measurement data are presented as the mean ± S.D., and *Student's t test* was used to test the significance of the differences of the compared samples. Non-normally distributed measurement data are presented as the median (IQR), and the *Mann–Whitney U test* was used for the analyses. Count data were analyzed by means of the *chi-square test*. *Receiver operating characteristic* (*ROC*) *curves* were generated with *SPSS*, and the areas under the curve (AUCs) were calculated. The best decision criteria were determined when the Youden index had the largest value (24). *P* < 0.05 was considered significantly different. Logistic regression analysis was used to identify the independent risk factors. All figures were drawn with GraphPad Prism (version 9.0; GraphPad Software, Chicago, IL, USA).

## Results

A total of 96 neonates diagnosed with NEC were included in the study, and 20 of the patients did not have complete parameter concentrations measured with a small sample of serum. Nineteen infants who had pneumoperitoneum diagnosed on abdominal radiographs before blood sampling were excluded, and 8 were diagnosed with other gastrointestinal diseases when the surgeries were performed. Therefore, 49 infants were enrolled in our study, with 23 in the surgical group and 26 in the medical group.

### Clinical features

The clinical features are shown in Table 1. There were no significant differences between the two groups in the baseline information, including the risk factors in pregnancy, delivery room handling, the medications used during pregnancy and NEC treatment (*P* > 0.05). The outcomes of hospitalization in the two groups showed no difference (*P* > 0.05), but the incidences of peritonitis, intestinal adhesion and sepsis in the surgical group were higher (*P* < 0. 05).

**Table 1:** Demographics of the infants enrolled in the study.

	**Medical group (*n* = 26)**	**Surgical group (*n* = 23)**	***χ^2^*/*Z*/*t***	***P***
Male, % (n)	50.0 (13)	47.8 (11)	0.023	0.879
Admission age, M (IQR), d	5.71 (0.07, 12.23)	5.92 (1.75, 11.67)	0.010	0.920
GA, x ± S. D., w	34.27 ± 3.432	35.02 ± 3.240	−0.789	0.434
Birth weight, M (IQR), g	2, 200.00 (1, 565.00, 2, 717.50)	2, 250.00 (1, 420.00, 2, 970.00)	0.036	0.849
Cesarean section, % (n)	76.9 (20)	47.4 (18)	0.013	0.911
PROM, % (n)	19.2 (5)	13.0 (3)	0.039	0.843
Apgar 1 min, M (IQR)	9 (7, 10)	10 (9, 10)	1.915	0.166
Apgar 5 min, M (IQR)	10 (9, 10)	10 (10, 10)	1.280	0.258
Age at NEC, x ± S. D., d	11.24 ± 8.414	11.13 ± 8.756	0.044	0.965
Weight at NEC, x ± S. D., g	2, 249.62 ± 596.949	2, 366.09 ± 806.416	−0.579	0.565
Death, % (n)	3.8 (1)	8.7 (2)	0.012	0.913
Peritonitis, % (n)	0.0 (0)	69.6 (16)	26.856	0.001
Bowel stenosis, % (n)	7.7 (2)	21.7 (5)	0.987	0.321
Intestinal adhesion, % (n)	7.7 (2)	52.2 (12)	11.832	0.001
Shock, % (n)	0.0 (0)	13.0 (3)	1.700	0.192
PDA, % (n)	23.1 (6)	21.7 (5)	0.013	0.911
Perinatal asphyxia, % (n)	11.5 (3)	0.0 (0)	1.176	0.278
Anemia before NEC, % (n)	53.8 (14)	69.6 (16)	1.270	0.260
Sepsis, % (n)	53.8 (14)	91.3 (21)	8.391	0.004
Meningitis, % (n)	3.8 (1)	4.3 (1)	1.000	0.724

GA, gestational age; PROM, premature rupture of membranes > 18 h; NEC, necrotizing enterocolitis; PDA, patent ductus arteriosus.

### Abdominal signs and imaging

**Figure 1:**
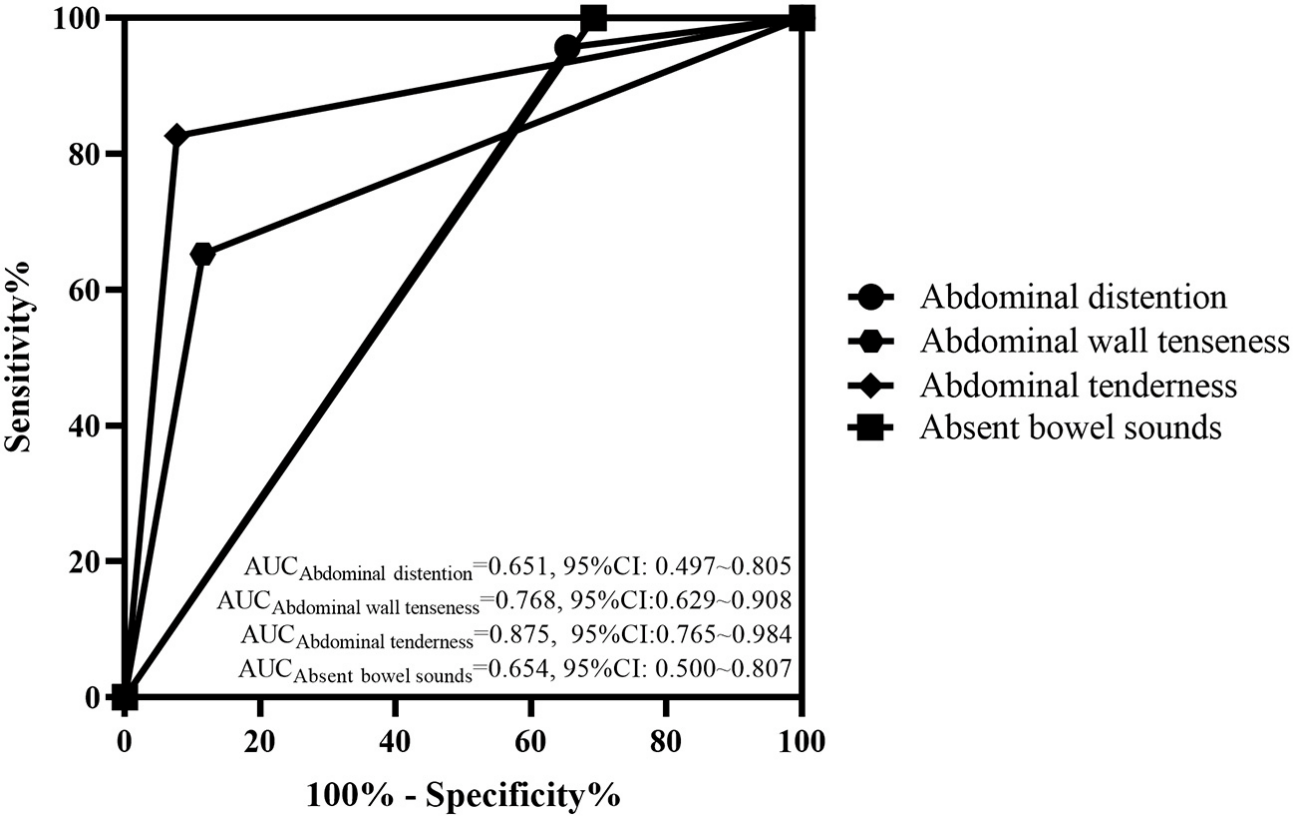
ROC curve of the abdominal signs in the two groups. The AUCs of abdominal distention, abdominal wall tenseness, abdominal tenderness and absent bowel sounds were 0.651 (95% CI: 0.497–0.805), 0.768 (95% CI: 0.629–0.908), 0.875 (95% CI: 0.765–0.984), and 0.964 (95% CI: 0.500–0.807), respectively.

The abdominal signs and imaging presentations when NEC was diagnosed in the two groups are shown in Table 2. There was a higher rate of abdominal distention, abdominal wall tenseness, abdominal tenderness and absent bowel sounds in the surgical group when NEC had occurred (*P* < 0.05) than in the medical group. There was no difference between the two groups regarding the signs of a thickened bowel wall, seroperitoneum and reduced peristalsis on ultrasound (*P* > 0.05). The typical abdominal imaging appearance of NEC on both X-ray and ultrasound and the presence of pneumatosis intestinalis and portal venous gas were also not different between the surgical group and the medical group (*P* > 0.05). The AUCs of abdominal distention, abdominal wall tenseness, abdominal tenderness and absent bowel sounds were 0.651 (95% CI: 0.497–0.805), 0.768 (95% CI: 0.629–0.908), 0.875 (95% CI: 0.765–0.984) and 0.654 (95% CI: 0.500–0.807), respectively. The AUCs are shown in Figure 1.

**Table 2:** Abdominal signs and imaging results of the infants in the two groups.

	**Medical group (*n* = 26)**	**Surgical group (*n* = 23)**	***χ^2^***	***P***
Abdominal distention, % (n)	65.4 (17)	95.7 (22)	5.146	0.023
Abdominal wall tenseness, % (n)	11.5(3)	65.2 (15)	15.131	0.001
Abdominal wall erythema, % (n)	0.0 (3)	13.0 (2)	1.700	0.192
Abdominal tenderness, % (n)	7.7 (2)	82.6 (19)	27.969	0.001
Absent bowel sounds, % (n)	69.2 (18)	100.0 (23)	6.355	0.012
Thickened bowel wall, % (n)	22.2 (4)^a^	20.0 (3)^b^	0.000	1.000
Seroperitoneum, % (n)	50.0 (9)^a^	73.1 (11)^b^	1.866	0.172
Reduced peristalsis, % (n)	5.6 (1)^a^	6.7 (1)^b^	0.000	0.710
Pneumatosis intestinalis, % (n)	69.2 (18)	52.2 (18)	1.496	0.221
Portal venous gas, % (n)	26.9 (7)	21.7 (5)	0.177	0.674

^a^n = 18; ^b^n = 15.

### Serum biomarkers

**Figure 2:**
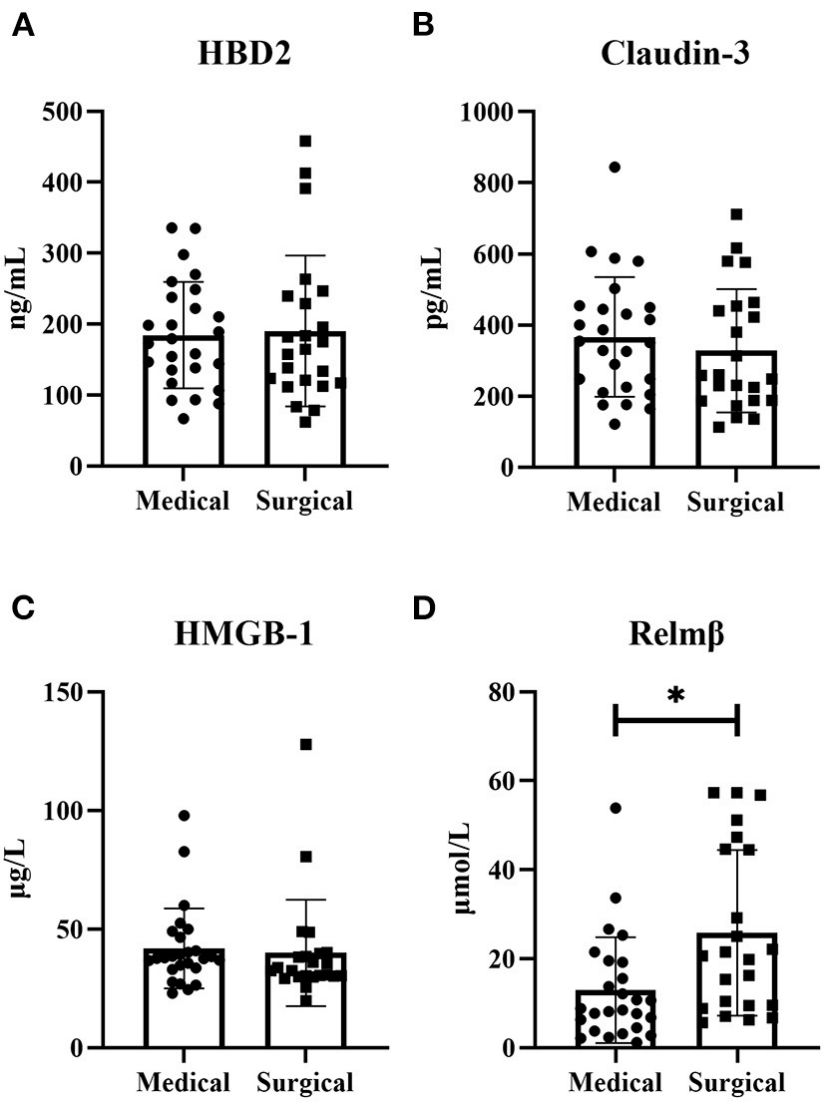
Concentrations of serum biomarkers in the two groups. The concentrations of HBD2, Claudin-3 and HMGB-1 showed no difference between the two groups (*P* > 0.05) **(A–C)**. The concentration of Relmβ in the surgical group was significantly increased compared with that in the medical group (*P* < 0.05) **(D)**.

**Figure 3:**
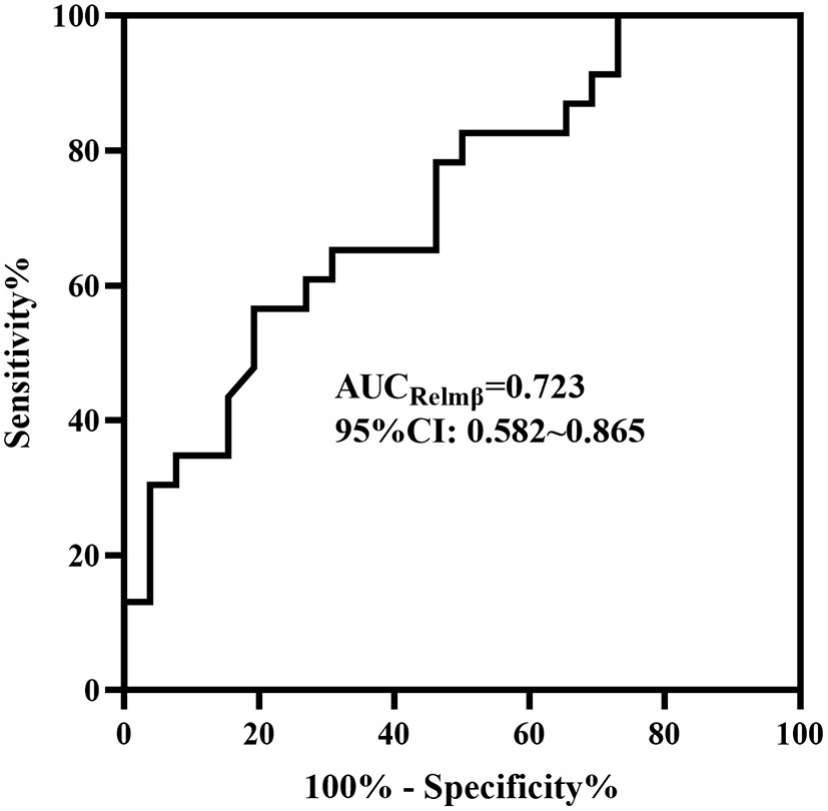
The ROC curve of Relmβ. The AUC of Relmβ was 0.723 (95% CI: 0.582–0.865).

In the medical and surgical groups, the concentrations of HBD2 [184.53 ± 75.093 vs. 190.54 ± 106.458], Claudin-3 [352.72 (221.68, 450.44) vs. 258.30 (187.70, 453.41)] and HMGB-1 [37.79 (33.43, 47.18) vs. 32.55 (30.27, 39.75)] showed no difference (*P* > 0.05). The concentration of Relmβ [8.66 (4.29, 19.28) vs. 20.65 (9.51, 44.65)] was significantly higher in the surgical group than in the medical group (*P* < 0.05), as shown in Figure 2. Further, the AUC of the *ROC curve* (Figure 3) was 0.723 (95% CI: 0.582–0.865), and we took 19.7 μmol/L for the decision criteria to obtain the largest Youden index with a sensitivity of 56.5% and specificity of 80.8%.

### Predictive scores

**Figure 4:**
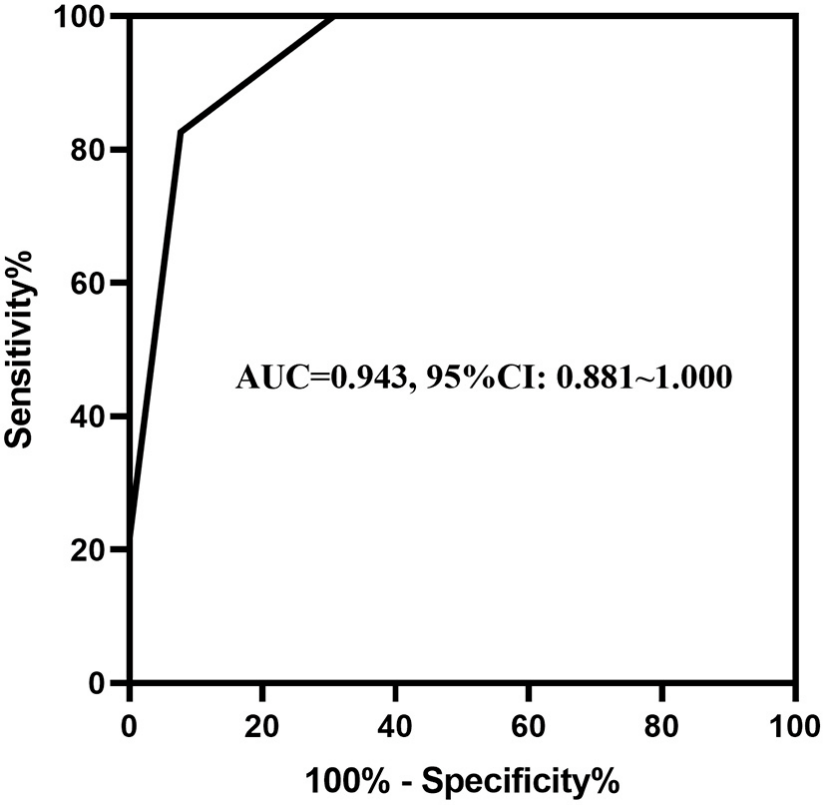
The ROC curve of abdominal signs combined with the serum biomarkers. The AUC of abdominal wall tenseness and abdominal tenderness combined with Relmβ concentration was 0.943 (95% CI: 0.891–1.000).

Abdominal distention, abdominal wall tenseness, abdominal tenderness, absent bowel sounds and Relmβ > 19.7 μmol/L were included in the multivariate stepwise logistic regression analysis and are shown in Table 3. Abdominal wall tenseness, abdominal tenderness and Relmβ > 19.7 μmol/L were independent risk factors for surgical NEC (*P* < 0.05), and the model showed a significant difference (*P* = 0.001). According to the regression coefficients, abdominal wall tenseness, abdominal tenderness and Relmβ > 19.7 μmol/L were scored as 1 point each to obtain the predictive scores. The scores [0.00 (0.00, 1.00) vs. 2.00 (2.00, 2.00)] in the two groups were highly significantly different (*P* < 0.05), and the *ROC curve* is shown in Figure 4. The AUC was 0.943 (95% CI: 0.891–1.000), and when the model score was 1.5 points, the sensitivity and specificity were 82.6 and 92.3%, respectively. Therefore, the model showed high predictive value in surgical decision-making when the score was ≥2 points.

**Table 3:** Multivariate analysis of predictors of the surgical timing.

**Variable**	**β**	**SE**	**Wald**	***P***	**OR**	**95% CI**
Abdominal wall tenseness	2.842	1.314	4.682	0.030	17.154	1.307–225.138
Abdominal tenderness	3.152	1.061	8.821	0.003	23.384	2.921–187.188
Relmβ > 19.7 μmol/L	2.732	1.274	4.596	0.032	15.365	1.264–186.761

## Discussion

NEC is a fatal disease during the neonatal period with high morbidity and mortality, especially for neonates who undergo surgery. To decrease the incidence of infection and shock along with infection and improve the prognosis after surgery, it is very important to master the surgical timing. Full-thickness intestinal necrosis without perforation has been reported to be the best surgical indication (5). It has been reported that the bacterial signaling receptor Toll-like receptor 4 seems to play an important role in the development of NEC especially in those preterm infants (25). However, no signaling molecules have been demonstrated to be clinically effective in reflecting the severity of necrosis. Therefore, a prospective cohort study was performed, and we found that serum Relmβ combined with the addition of abdominal signs can predict surgical timing in NEC neonates.

Khalak revealed that a standardized physical examination score composed of abdominal girth, abdominal erythema, abdominal discoloration, abdominal tenderness, absent bowel sounds, capillary refill and abdominal wall tenseness is sensitive and specific for predicting the need for surgery when the scores are higher than three points (26), and these results are consistent with our findings. However, it is rare that all the above abdominal signs appear in one patient, which makes the physical examination lack specificity in the diagnosis of peritonitis (27). Therefore, it is essential to combine these physical examination factors with other biomarkers that can reflect intestinal inflammation to guide decision-making.

Previous studies have shown that some biomarkers, including intestinal and liver fatty acid-binding protein, trefoil factor-3, fecal calprotectin, HBD2, HMGB-1, Claudin-3 and Relmβ, can be potential biomarkers for diagnosing NEC, and some of these markers may even indicate the severity of NEC to some extent (16–18, 28, 29). In our study, we detected the serum concentrations of HBD2, HMGB-1, Claudin-3 and Relmβ and found that a concentration of Relmβ higher than 19.7 μmol/L in infants with NEC may indicate that the infants need surgery. Relmβ is a bactericidal protein that is increasingly secreted in intestinal inflammation (30); it can bind to lipids on the surface of gram-negative bacteria and destroy the cell membranes to exert a bactericidal effect (20). Luo found that the serum Relmβ level in neonates with NEC was significantly higher and was correlated with the severity of NEC (22), which also supports our result. The relationships between NEC and HBD2 and HMGB-1 in the feces and Claudin-3 in the urine have been clearly demonstrated, but the value of these parameters in the serum remains unknown. It has been reported that HBD2 ranges with age (31), and serum HMGB-1 also increases in other neonatal diseases, such as persistent pulmonary hypertension (32), hypoxic-ischemic encephalopathy (33) and biliary atresia (34). Therefore, many factors can affect the concentrations of these biomarkers in serum. In our study, HBD2, HMGB-1 and Claudin-3 showed no difference between the groups, and the values of these biomarkers for predicting the surgical timing need further exploration.

In our study, abdominal signs and serum biomarkers were combined, and we found that when the score was ≥2 points, the model showed high value for guiding surgical decision-making. Because patients who had intestinal perforation were excluded and because all the infants enrolled in the surgical group nearly reached the optimal surgical indication, the scoring system can be used to predict the timing of surgery more accurately before intestinal perforation. As the center of the Neonatal Emergency Transport System in southwestern China, our department serves neonates in the provinces of Chongqing, Sichuan Guizhou and Yunnan. In 2019 and 2020, 19,381 neonates were admitted to our department and 5,294 to neonatal intensive care unit. In the 2 years, 918 infants were diagnosed with NEC, and among them 304 underwent surgeries. Many of the cases of NEC in our study were late preterm and term infants for most infants admitted to our department were in larger gestation. Thus, our study can provide clinical guidance for surgical decision-making of these infants in Southwest China.

However, there were still some limitations in our study. It remained unknown if there were also full-thickness necrosis in intestinal wall of these infants in medical group and how much intestinal wall had full-thickness necrosis in surgical group. The extent of necrosis should be evaluated by the surgeons during surgery. In addition, a previous study showed that a thickened bowel wall, seroperitoneum and reduced peristalsis on ultrasound represent continuous changes that reflect the severity of NEC (35). However, in our study, for a few infants undergoing ultrasound, dynamic monitoring was not performed, and ultrasound parameters were not included. Moreover, because the value of HBD2, HMGB-1 and Claudin-3 in the serum remains unknown, clinical trials with a larger sample size and dynamic monitoring are needed.

## Conclusion

Isolated abdominal signs including abdominal distention, abdominal wall tenseness, abdominal tenderness and absent bowel sounds have low to moderate value in the prediction of surgical timing-point. The concentration of serum Relmβ > 19.7 μmol/L has a moderate value in the surgery-decision making of NEC when considered in isolation; a predictive model combining abdominal wall tenseness, abdominal tenderness and Relmβ > 19.7 μmol/L showed high predictive value when the score was ≥2 points. In a summary, serum Relmβ concentration combined with abdominal wall tenseness and abdominal tenderness may reliably predict the need for surgery in NEC neonates. More potential biomarkers and new imaging methods for predicting the timing of NEC surgery need further research.

## Data availability statement

The raw data supporting the conclusions of this article will be made available by the authors, without undue reservation.

## Ethics statement

The studies involving human participants were reviewed and approved by Ethics Committee of the Children's Hospital Affiliated to Chongqing Medical University. Written informed consent to participate in this study was provided by the participants' legal guardian/next of kin.

## Author contributions

X-CL contributed to the collection of clinical data, the analysis and interpretation of the data, and the drafting and final approval of the manuscript. LG contributed to the collection of clinical data and serum samples, sample detection, and drafting of the manuscript. K-RL and X-YH contributed to the collection of the serum samples and the sample detection. Y-JS contributed to the collection of the serum samples. L-QL supervised the project, contributed to the conception and design of the study, to the analysis and interpretation of the data, and to the critical revision and final approval of the manuscript. All authors made substantial contributions to the study and manuscript.

## Conflict of interest

The authors declare that the research was conducted in the absence of any commercial or financial relationships that could be construed as a potential conflict of interest.

## Publisher's note

All claims expressed in this article are solely those of the authors and do not necessarily represent those of their affiliated organizations, or those of the publisher, the editors and the reviewers. Any product that may be evaluated in this article, or claim that may be made by its manufacturer, is not guaranteed or endorsed by the publisher.
